# Combined Trochleaplasty, Lateral Retinacular Lengthening, and Medial Patellofemoral Ligament Reconstruction Using Quadriceps Tendon

**DOI:** 10.1016/j.eats.2025.103977

**Published:** 2025-11-02

**Authors:** Julian Mehl, Sebastian Siebenlist, Lukas Willinger

**Affiliations:** Department of Sports Orthopaedics, Technical University Munich, Munich, Germany

## Abstract

Chronic and recurrent patellar instability frequently is associated with trochlear dysplasia, lateral retinacular tightness, and medial patellofemoral ligament (MPFL) insufficiency. Combined surgical correction addressing all pathologic factors is critical to restoring patellar stability. This Technical Note presents a detailed and reproducible surgical technique combining deepening trochleaplasty, lateral retinacular lengthening, and MPFL reconstruction using a quadriceps tendon autograft. Trochleaplasty is performed using a thin-flap technique to create a new, stable trochlear groove. Lateral retinacular lengthening is carried out via a stepwise Z-plasty, and an anatomic MPFL reconstruction is accomplished with a partial-thickness quadriceps tendon graft without patellar bone tunnels. This comprehensive approach aims to restore patellar tracking, reduce pain, and prevent recurrent dislocations. The described technique offers a systematic strategy for treating complex patellar instability while preserving joint congruency and minimizing complications.

Patellar instability is a multifactorial pathology frequently encountered in young, active populations, with an incidence of 5.8 to 7.0 per 100,000 person-years.^1^^,^^2^ Trochlear dysplasia is the leading anatomical risk factor for chronic patellofemoral instability and present in the majority of revision cases after medial patellofemoral ligament (MPFL) reconstruction.^3^^,^^4^ Medial soft-tissue insufficiency, particularly MPFL disruption, is identified in nearly all patients after primary dislocation, and lateral retinacular tightness further contributes to maltracking and instability.^5^

Isolated procedures addressing only 1 pathologic factor have shown limited success in patients with combined anomalies.^5^ Trochleaplasty procedures realign the trochlear groove and are indicated for high-grade trochlear dysplasia.^6^^,^^7^ MPFL reconstruction restores medial restraint, whereas lateral retinacular lengthening addresses lateral soft-tissue tension.^8^^,^^9^

This Technical Note details a stepwise surgical technique combining trochleaplasty, lateral retinacular lengthening, and MPFL reconstruction using a quadriceps tendon autograft to comprehensively treat recurrent patellar instability associated with high-grade trochlear dysplasia and lateral tightness (Table 1).

**Table 1:** Pearls and Pitfalls of the Described Surgical Technique

Pearls	Pitfalls
Thin-flap trochleaplasty minimizes cartilage injury	Making the trochlear groove too deep may cause cartilage damage and impingement
Z-plasty lengthening preserves vascularity	Excessive lateral release may cause medial instability
Use of quadriceps graft avoids bone tunnels in the patella	Overtensioning the medial patellofemoral ligament graft can lead to medial patellar overload

## Surgical Technique

### Indication and Diagnosis

**Fig 1:**
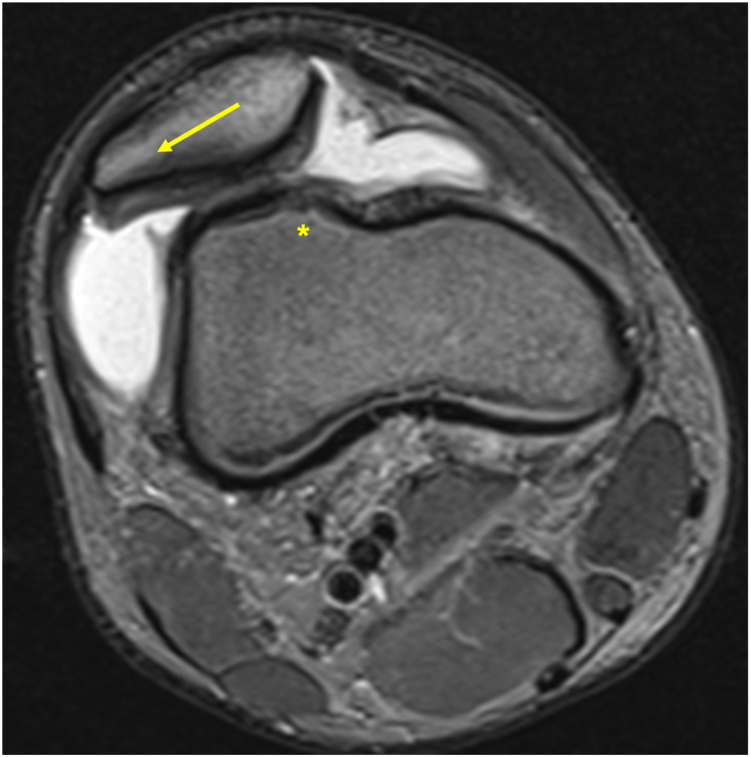
Axial magnetic resonance imaging of a right knee with chronic patellofemoral instability, lateral maltracking of the patella (yellow arrow) with tightness of the lateral retinaculum, medial patellofemoral ligament insufficiency and high grade trochlea dysplasia (∗).

The indication for combined surgery is determined on the basis of clinical examination and imaging, including anteroposterior and true lateral radiographs, long-leg radiographs, magnetic resonance imaging axial sequences, and computed tomography scans for assessment of trochlear dysplasia (Dejour classification), tibia tubercle-trochlea groove distance, and patellar tilt (Fig 1).

Clinical examination includes the detection of patella apprehension in different knee flexion angles. Apprehension in knee flexion greater than 30° should raise suspicion for trochlea dysplasia. Trochlea dysplasia also is suspected when a positive J sign is present during active flexion and extension of the knee. Surgical intervention is indicated in cases of recurrent instability with high-grade trochlear dysplasia (types B–D), lateral retinacular contracture, and MPFL insufficiency.

### Patient Positioning and Setup

The patient is placed supine under general anesthesia. A lateral post is positioned at midthigh and a foot roll is used to allow free knee motion. A tourniquet is applied and inflated before surgery commences. Prophylactic antibiotics are administered.

### Diagnostic Arthroscopy

Standard anterolateral and anteromedial portals are created. Diagnostic arthroscopy is performed with a special focus on patellofemoral cartilage lesions, patellar tracking and the shape of the trochlea. An additional superolateral portal is recommended for better visualization of the dynamic patellofemoral alignment. If significant further intra-articular pathologies like cartilage or meniscus lesions are detected, they are addressed arthroscopically before proceeding.

### MPFL Reconstruction With Quadriceps Tendon

**Fig 2:**
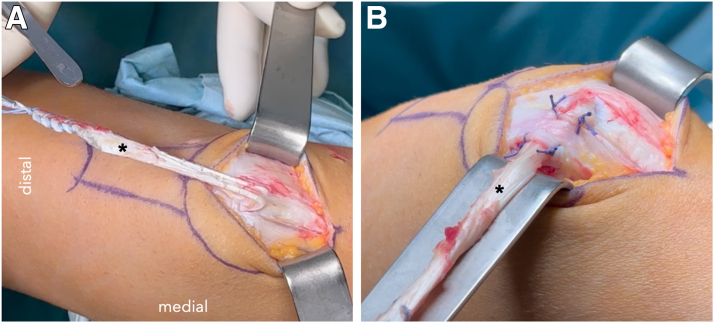
View from medial side from harvesting and rerouting of the quadriceps tendon for medial patellofemoral ligament reconstruction of a right knee. (A) The thin superficial layer of the quadriceps tendon with a length of 11 cm is harvested and detached proximally (∗). The proximal end is secured with a nonabsorable FiberWire suture in whipstitch technique. The distal patellar attachment is preserved. (B) The graft is flipped medially, routed underneath a soft-tissue layer and secured with 4 absorbable sutures to the medial patella periosteum.

The superficial central portion of the quadriceps tendon is harvested as a partial-thickness graft (3 mm) measuring approximately 10 mm wide and 10 to 11 cm long, leaving its distal attachment intact. The tendon is flipped medially and routed under a periosteal tissue layer of the medial patella.^10^ It is secured with 4 knots with absorbable sutures (VICRYL 1-0; Ethicon, a Johnson & Johnson Company; Fig 2).

At the medial border of the patella, the second medial layer is sectioned and the MPFL graft routed posteriorly. The location of the femoral attachment is identified in true lateral fluoroscopy and a 6-mm drill tunnel is created. The free end of the graft is routed through the tunnel and fixed at the femoral MPFL insertion site (between the medial epicondyle and the adductor tubercle) using an 6- × 20-mm biocomposite interference screw (Arthrex) under fluoroscopic guidance. Tensioning is performed at 30° of knee flexion with neutral patellar alignment (Table 2).

**Table 2:** Advantages and Disadvantages of the Described Surgical Technique

Advantages	Disadvantages
Comprehensive correction of instability factors	Technically demanding
Preservation of cartilage vitality	Longer surgical time
No patellar drill tunnels	Greater rehabilitation demands
Low donor site morbidity	Risk of postoperative stiffness if not properly rehabilitated

### Lateral Retinaculum Lengthening

**Fig 3:**
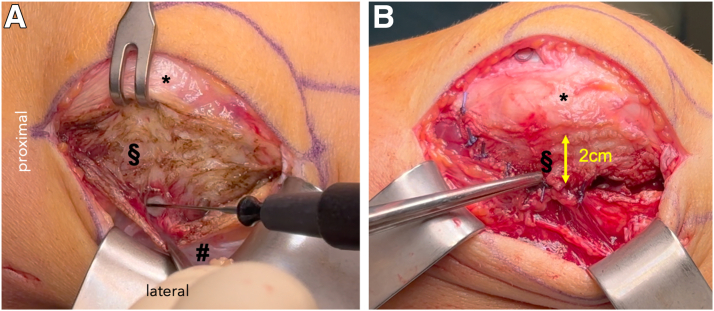
View from the lateral side onto a right knee, with the proximal end on the left side. (A) Lengthening of the lateral retinaculum with a Z-plasty technique. The superficial layer (#) is held down by the forceps while the deep layer (§) is incised. The retractor retracts the patella medially (∗). (B) Closure of the lateral retinaculum with lengthening of approximately 2 cm with absorbable VICRYL No. 1 sutures.

Surgery is performed through a single midline skin incision of about 8 to 10 cm. Before trochleaplasty, attention is turned to the lateral retinaculum. A Z-plasty is performed by creating longitudinal incisions in both the superficial oblique and deep transverse fibers approximately 2 cm apart (Fig 3). Controlled lengthening is achieved by shifting the flaps and suturing them in an elongated configuration with absorbable sutures (e.g., VICRYL 2-0; Ethicon). This step decreases lateral tethering forces and improves patellar tilt without overt lateral release, preserving patellar vascularization.

### Trochleaplasty

After the incision of the lateral retinaculum, the patella is everted medially to expose the trochlea. The trochlea shape and the position of the groove is analyzed and the new orientation of the neotrochlea is planned. The height of the groove is aligned with the anterior cortex of the femoral shaft. This reference guarantees a complete resection of the osseous bump at the proximal trochlea which is a typical feature of high-grade dysplasia.

**Fig 4:**
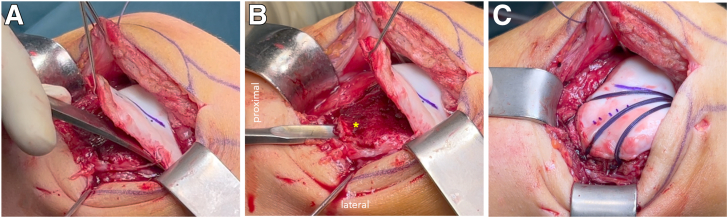
View from lateral at a right knee. (A) A thin osteochondral flap is raised proximally from the dysplastic trochlea using thin osteotomes. (B) A new trochlea groove is created with a gouge (∗) while the osteochondral flap is held superiorly with a retractor. (C) The osteochondral flap is fixed with VICRYL sutures and PushLock anchors. One anchor is inserted near the femoral notch while three anchors are fixing the VICRYL sutures at the proximal end of the trochlear (medial, central, lateral).

A thin osteochondral flap (approximately 3 mm) is raised from the dysplastic trochlea using thin osteotomes and a curve chisel (Fig 4A).^6^ A new trochlear groove is created by removing subchondral bone beneath the flap, with a gouge and burr focusing on deepening the central sulcus and elevating the lateral facet (Fig 4B). The osteochondral flap is then molded into the newly created groove and fixed using bioabsorbable anchors (e.g., PushLock; Arthrex). One central anchor is placed centrally in the distal trochlea and absorbable No. 1 VICRYL sutures are tensioned peripherally with three anchors at the proximal end of the trochlea, ensuring an anatomic trochlear morphology (Fig 4C). In order to apply planar compression on the cartilage without risk of cutting through, it is recommended to tension at least three parallel VICRYL sutures (at least 3) to each peripheral anchor. After fixation of the flap, the joint capsule is closed and the lateral retinaculum is lengthened as described previously (Table 3, Video 1).

**Table 3:** Special Instruments Used for the Surgery

Instrument	Purpose
High-speed burr	Trochlear groove deepening
Bioabsorbable anchors (e.g., PushLock)	Osteochondral flap fixation
Interference screw	Medial patellofemoral ligament graft femoral fixation

### Closure

Skin closure is completed in layers. A sterile compressive dressing is applied, and a hinged knee brace locked in extension is fitted.

### Postoperative Rehabilitation

Postoperatively, weight-bearing is allowed as tolerated with the brace locked in extension for 6 weeks. Range of motion is gradually advanced:

o Weeks 1–2: extension/flexion (E/F): 0° to 30°
o Weeks 3–4: E/F: 0° to 60°
o Weeks 5–6: E/F: 0° to 90°

Quadriceps activation is encouraged immediately. Return to low-impact activities begins at 3 months, whereas pivoting sports are delayed until 6 to 9 months postoperatively.

## Discussion

Comprehensive treatment of patellar instability demands a tailored approach to address bony and soft-tissue abnormalities.^3^^,^^4^^,^^11^ Isolated MPFL reconstruction may fail if significant trochlear dysplasia or lateral retinacular tightness is left uncorrected.^4^ Conversely, trochleaplasty alone may not suffice without restoring medial stability.^6^

The thin-flap trochleaplasty technique preserves cartilage viability while correcting trochlear morphology.^7^ Lateral retinacular lengthening prevents overconstraint and patellar maltracking, which can result from simple lateral release.^8^^,^^9^ MPFL reconstruction using quadriceps tendon offers an anatomic graft with minimal donor site morbidity, avoiding the risk of patellar fractures associated with bone tunnels.^10^^,^^12^

However, combined procedures are technically demanding and should be reserved for high-volume centers. Future studies with long-term follow-up are needed to validate the clinical superiority of this integrated approach.

## Declaration of Generative AI and AI-Assisted Technologies in the Writing Process

During the preparation of this work, the authors used ChatGPT (ChatGPT-4-turbo, OpenAI) in order to assist with language refinement and structuring of the manuscript. After using this tool, the authors reviewed and edited the content as needed and takes full responsibility for the content of the publication.

## Disclosures

All authors (J.M., S.S., L.W.) declare that they have no known competing financial interests or personal relationships that could have appeared to influence the work reported in this paper.

## Supplementary Data

Video 1 Shown is a step-by-step approach to a single incision combined trochleaplasty, lengthening of the lateral retinaculum, and MPFL reconstruction using quadriceps tendon. First the MPFL quadriceps graft is harvested and routed medially underneath a soft tissue layer of the patella. It is secured with VICRYL sutures to the patella. It is routed posteriorly in the 2^nd^ medial layer and fixed with a 6- × 20-mm biocomposite interference screw. A lateral retinaculum lengthening (Z-plasty) is performed by creating longitudinal incisions in both the superficial oblique and deep transverse fibers approximately 2 cm apart. A thin osteochondral flap is raised from the dysplastic trochlea using thin osteotomes and a curve chisel. A new trochlear groove is created by removing subchondral bone beneath the flap, with a gouge and burr focusing on deepening the central sulcus and elevating the lateral facet. The osteochondral flap is then molded into the newly created groove and fixed using bioabsorbable anchors (e.g., PushLock, Arthrex) (MPFL, medial patellofemoral ligament.)

## References

[1] Arendt E.A., Dejour D. (2013). Patella instability: Building bridges across the ocean a historic review. Knee Surg Sports Traumatol Arthrosc.

[2] Fithian D.C., Paxton E.W., Stone M.L. (2004). Epidemiology and natural history of acute patellar dislocation. Am J Sports Med.

[3] Dejour H., Walch G., Nove-Josserand L., Guier C. (1994). Factors of patellar instability: An anatomic radiographic study. Knee Surg Sports Traumatol Arthrosc.

[4] Feucht M.J., Mehl J., Forkel P. (2020). Failure analysis in patients with patellar redislocation after primary isolated medial patellofemoral ligament reconstruction. Orthop J Sports Med.

[5] Shah J.N., Howard J.S., Flanigan D.C., Brophy R.H., Carey J.L., Lattermann C. (2012). A systematic review of complications and failures associated with medial patellofemoral ligament reconstruction for recurrent patellar dislocation. Am J Sports Med.

[6] von Knoch F., Bohm T., Burgi M.L., von Knoch M., Bereiter H. (2006). Trochleaplasty for recurrent patellar dislocation in association with trochlear dysplasia. A 4- to 14-year follow-up study. J Bone Joint Surg Br.

[7] Blond L., Schottle P.B. (2010). The arthroscopic deepening trochleoplasty. Knee Surg Sports Traumatol Arthrosc.

[8] Liu C., Duan G., Niu Y. (2018). Lateral retinaculum plasty instead of lateral retinacular release with concomitant medial patellofemoral ligament reconstruction can achieve better results for patellar dislocation. Knee Surg Sports Traumatol Arthrosc.

[9] Migliorini F., Maffulli N., Eschweiler J., Quack V., Tingart M., Driessen A. (2021). Lateral retinacular release combined with MPFL reconstruction for patellofemoral instability: A systematic review. Arch Orthop Trauma Surg.

[10] Fink C., Veselko M., Herbort M., Hoser C. (2014). MPFL reconstruction using a quadriceps tendon graft: Part 2: Operative technique and short term clinical results. Knee.

[11] Nomura E. (1999). Classification of lesions of the medial patello-femoral ligament in patellar dislocation. Int Orthop.

[12] Peter G., Hoser C., Runer A., Abermann E., Wierer G., Fink C. (2019). Medial patellofemoral ligament (MPFL) reconstruction using quadriceps tendon autograft provides good clinical, functional and patient-reported outcome measurements (PROM): A 2-year prospective study. Knee Surg Sports Traumatol Arthrosc.

